# The Effect of Conflict Resolution Training on Children’s Behavioral Problems in Shiraz, Southern Iran: A Randomized Controlled Trial

**Published:** 2014-07

**Authors:** Sara Soleimani, Farkhondeh Sharif, Arash Mani, Sareh Keshavarzi

**Affiliations:** 1 Department of Mental Health and Psychiatric Nursing, School of Nursing and Midwifery, Shiraz University of Medical Sciences, Shiraz Iran;; 2 Community Based Psychiatric Care Research Center, Department of Mental Health and Psychiatric Nursing, School of Nursing and Midwifery, Shiraz University of Medical Sciences, Shiraz, Iran;; 3 Department of Psychiatry, Shiraz University of Medical Sciences, Shiraz, Iran;; 4 Department of Epidemiology, School of Health and Nutrition, Shiraz University of Medical Sciences, Shiraz, Iran

**Keywords:** Communication, Child Behavior Disorders, Marital Conflict

## Abstract

**Background: **There is evidence that marital problems can contribute to child behavior problems. In fact, the way that parents solve their conflicts, such as aggression, physical violence, and poor communication skills, can eventually culminate in aggression and emotional problems in children. This study aimed to evaluate the effectiveness of conflict resolution training on children’s behavioral problems in a sample of Iranian couples.

**Methods:** This randomized controlled trial study was carried out on the couples who referred to counseling centers in Shiraz, Iran. In this study, 56 couples were selected through convenience sampling and assigned to an intervention and a control group. The intervention group received 10 sessions of communication skills training. All the participants filled out conflict resolution questionnaire and Child Behavior Problem Checklist (CBCL). To analyze the data we used the SPSS statistical software (version 16), using repeated measurement test, paired *t*-test, and independent *t *test.

**Results:** In this study, no statistically significant differences were found between the two groups regarding the demographic characteristics. Also, no significant difference was observed between the two groups regarding the mean score of child behavior problems. Besides, a significant difference was found in the intervention group’s mean score of marital conflict in post-test compared to the pre-test; however, no such trend was observed in the control group.

**Conclusion:** Conflict resolution skill training was effective in reducing marital conflict. Also, it showed a slight reduction in the score of child behavior problems after the intervention. But this reduction wasn’t statisticaly significant.

**Trial Registration Number: **IRCT201109112812N2

## Introduction

Marriage has historically been one of the most important events in people’s lives and each person can decide who and when to marry. On the other hand, the mental health of the family, as the center of love and peace, is highly important since this constitution can help the growth of talents among its members and any damage to it will have irreversible effects on the future generation.^1^

Since people have different perceptions, viewpoints, and goals, interpersonal conflicts are sometimes unavoidable. If couples do not deal with conflicts in a constructive way, it may eventually lead to their separation.^2^

Divorce also has adverse effects on the children. They may suffer from symptoms, such as fear of being abandoned, confusion, sadness, anxiety, loneliness, and anger. Moreover, academic failure, behavioral and emotional problems, and low self-esteem in interpersonal relationships are more prevalent among the children of divorce, regardless of age and sex.^3^^,^^4^

Other researchers conducted a study to assess the effect of sexual skill training on marital satisfaction. In this program, 12 married female attended five two-hour sexual training classes. This study indicated that sexual skill training could play a significant role in increment of marital satisfaction.^5^

HosseinKhanzadeh and Yeganeh carried out a research to evaluate the effects of life skill training on marital satisfaction. The study findings indicated that life skill training had positive effects on marital adjustment level by improving their communication.^6^

In another study, Arfaie et al. stated that marital interaction pattern could predict the child’s behavioral problem. They also showed that children’s conduct problems could spring from their parents’ inappropriate ways of solving their marital conflicts.^7^

Furthermore, Faircloth and Cummings evaluated parent education program for preventing the negative effects of marital conflicts. In that study, 55 couples were randomly assigned to either an immediate treatment (n=41) or a six-month wait listed control group (n=14), with assessments being performed at pretest, posttest, after 6 months, and after 1 year. The results indicated that these programs could improve the parents’ knowledge towards marital conflicts. Also, the couples announced conducting less oppositional behavior in front of their children and having more effective conflict resolution in their relationships.^8^

Some other studies have reported the effects of marital conflicts on many aspects of child performance. Researchers have shown the effects of marital conflicts on the children’s internalized (e.g. depression) and externalized (e.g. aggression) behaviors as well as their social performance. The children sensitive to conflicts have lower thresholds for distress and aggression which increases the risk of developing adjustment problems. Moreover, the couples’ verbal conflicts may destroy the child-parent relationships and enhance the behavioral problems in children.^8^^,^^9^ Furthermore, increased family conflicts, low socioeconomic status, and sudden death of a parent can increase the risk of childhood and adolescence depression.^10^

In Turkey, a study was done to determine the effects of communication training on improving conflict resolution skills in 40 couples who had obtained the worst scores in the marital conflict questionnaire. The results showed that after participation in the program, the intervention group obtained higher scores compared to the control group. Similar results were also obtained in a follow-up study that was performed 3 and 6 months later.^11^

Halford and Markman did a review study on the effectiveness of couple relationship education. They concluded that a variety of variables, including poor communication skills, could affect the relationship satisfaction and stability and the couple relationship education could be effective for the couples who suffered from marital problems.^12^

Moreover, many studies have revealed the positive effects of teaching problem solving skills, anger management, and communication skills on reducing the marital conflicts and increasing marital satisfaction.^13^^,^^14^

Not all forms of marital conflicts lead to aggression. Children respond aggressively to destructive behaviors and negative emotional responses.^15^ Negative parenting and wrong management of marital conflicts leave the children feeling distressed even when the parents do maintain positive parenting relations with their children. Thus, it seems that interventions need to be broadened to include training for effective marital problem solving, communication, anger management skills, and parenting skills.^16^ These interventions also help couples understand and identify which aspects of their behavior are detrimental to child adjustment early in their relationship.^8^ The present study aims to evaluate the effectiveness of communication skill training in the children’s behavioral and performance problems in a sample of Iranian couples.

## Materials and Methods

Study Design and Population

The present study was a randomized controlled trial. Based on previous studies and research objectives, using Med Calc software, and considering confidence interval of 95% and power of 80%, a 66-couple sample size was determined for the study. The couples were selected among those who had referred to the counseling centers of Shiraz, southern Iran during 2011 using convenience sampling. So, all the couples who referred to the consulting centers, and had the necessary inclusion criteria were enrolled in the study. Then, they were divided into an intervention (n=33 couples) and a control group (n=33 couples), using balanced block randomization. In this study, eight couples dropped out of the intervention group for refusing to continue their cooperation, having small children, their husbands’ occupation, inability to attend the training classes, etc. Also, 2 couples dropped out of the control group for the same reasons listed above. Then, 56 couples remained in the study. (25 couples in the intervention group and 31 in the control group).

The inclusion criteria of the study were obtaining a score of 60 or higher in the marital conflict questionnaire, aged 6-12 year old children, not being in the process of divorce, not suffering from psychological disorders, and willingness to participate in the study. The participants filled out marital conflict questionnaire, Child Behavior Problem Checklist (CBCL), and demographic data form at the begining of the study. 

Approximately one month after agreeing to participate in the study, the intervention group couples referred to the consultation center to participate in the educational program. For each of the three intervention groups, 10 training sessions, each lasting for 1.5 hours, were held twice a week on Saturdays and Wednesdays for 5 weeks (morning and evening). The program was presented in groups of up to 11 couples. However, the children were not involved in these sessions. The couples who were not able to attend a meeting could participate in the other training sessions with the same subject which were held at different times. The marital conflict and CBCL were filled out again by the couples in the final session. The participants were also required to refer to the center again one month after the intervention to fill out the questionnaires again. One month after the intervention, the questionnaires were completed by the wait-listed control group, as well. At the end of the program, the communication skill training booklet was given to the control group.

This study was approved by the Ethic Committee of Shiraz University of Medical Sciences in May 2011 (No. 5460). The anonymity of the participants was preserved and no financial burden was imposed on them. All the participants signed the written informed consent before entering to the study.

Measures

Marital Conflict Questionnaire

Marital conflict questionnaire was developed by Barati and Sanayi in Iran.^17^ This questionnaire contains 42 questions for assessing marital conflict and evaluates seven aspects of marital conflicts; i.e., reduction of cooperation, reduction of sexual relationships, increase of emotional reactions, increase in attracting the children’s support, enhancment of personal relationships with one’s family, reduction of personal relationship with one’s partner’s family and friends, and separation their financial affairs. This questionnaire can also be used by counselors and other clinical experts for assessing marital conflicts.

Each item in the marital conflict questionnaire has five response options and a score of 1-5 is assigned to each option. Thus, the maximum and minimum total score of the questionnaire are 210 and 42, respectively. Each of the 42 items of this questionnaire corresponds to one of the seven mentioned aspects of marital conflicts. In this questionnaire, a higher score implies more conflict.^17^ The content validity of the questionnaire was confirmed by several professors of Shiraz University of Medical Sciences. Besides, its Cronbach’s alpha (internal correlation) was examined by conducting a pilot study on 60 women who were working as nurses in Zahra Hospital in Shiraz. After two weeks, the reliability of the questionnaire was assessed using the test-retest method and Cronbach’s alpha of 86% was obtained.

Child Behavior Problem Checklist (CBCL)

Child Behavior Problem Checklist developed by Achenbach was used in this study.^18^ The original version of CBCL includes 113 questions. However, after translation and content validity review in Iran, some questions were deleted because of cultural and social reasons and other restrictions. Also, based on a pilot study, several behaviors that were mentioned by some parents were added to the final scales.^19^ The items that were omitted from the questionnaire were drinking alcohol without parents’ approval, playing with one’s own sexual organs in public, playing with one’s sexual organs too much, running away from home, sexual problems, getting hurt a lot, being accident-prone, stealing from one’s own home, stealing from others, thinking about sex too much, smoking, chewing or sniffing tobacco, drug abuse, deliberately harming one-self or trying to commit suicide, being afraid of certain animals, situations, or places other than school, hearing non-present sounds or voices, preferring to be alone than with others, feeling dizzy or lightheaded, being overweight, preferring to be with older kids, setting fire, showing off or clowning, physical problems without known medical causes such as pain (not stomachache or headache), headache, nausea, vomiting, rashes or other skin problems, storing too many things that one does not need, thumb-sucking, wetting one-self during the day, truancy, escaping from school, enjoying less often, and sudden change in moods or feelings.^18^ The items that were added to the checklist were being adventurer, being persnickety, being paranoid, and being in low mood.^19^

The behavioral scale utilized in the present study included 79 questions on the parents’ feedback about their child’s behavior as a result of observing the child’s behavior. Cronbach’s alpha of the entire inventory was obtained as 80%.^19^ In addition, the content validity of the scale was verified by the help of the experts in the field of psychology. The content validity of this scale was further confirmed by comparing the content of this scale with other behavioral measures, such as Koy and Kanzer behavioral measures. This questionnaire measures six factors in child behavior, including aggression, immaturity of behavior, social withdrawal, destructive behavior, obsessive compulsive disorder, and anxiety. The reliability and validity of this scale have been confirmed by Samani in Shiraz, Iran.^19^

Treatment Process

Because the couples of the intervention group worked and had to take care of their children, they were not able to participate in the training classes at the same time. Therefore, based on when they could attend the classes, they were divided into three groups. The training classes were held twice a week at specified times for each group:

In each training sessions, 11 couples participated and activities such as information presentation, role playing, and homework were performed based on the skills. After each activity, the couples were given homework to transfer what was gained from the session into real life situations. What follows are the intended skills in the training sessions: 

First group: Saturdays and Wednesdays: 9-10:30 A.M

Second group: Saturdays and Wednesdays: 4-5:30 P.M

Third group: Saturdays and Wednesdays 6:30-8 P.M

The content of the training sessions was obtained from an educational program that was used in Iran by Fathi-Lavasani.^20^ The training provided knowledge about the side effects of inappropriate conflict resolution and how it affects wives, husbands, and children. Skills about healthy communication, active listening, assertiveness, how to cope with anger, and conflict resolution were also included in the training (table 1).

**Table 1:** Content of the intended skills in training sessions

**Number of sessions**	**Content of the sessions**
First session	The participants and the researcher met and became familiar with each other. The importance of communication skills in solving marital problems was explained.
Second session	Communication skills, importance of communication in marital relationship, and inappropriate way of communication were explained.
Third session	The participants were trained how to develop the ability of talking and active listening and learned about the unhealthy listening ways.
Forth session	The participants were explained about anger, its nature, and its causes.
Fifth session	The participants were trained on how to develop the ability of anger management and recognize signs of anger as well as anger provocative situations.
Sixth session	The participants were trained on how to gain the ability of relaxing their body and mind after getting angry.
Seventh session	The participants were trained on how to express anger and learned about the assertiveness skills.
Eighth session	The participants were taught on how to gain the ability of dealing with their wife or husband’s anger.
Ninth session	The participants were provided with information about conflict, its reasons, and different styles of solving it.
Tenth session	The participants were trained on how to develop cooperative conflict resolution skills with their partners. At the end of the sessions, the study participants shared their feeling about the workshop and evaluated the process.

## Results

**Figure 1:**
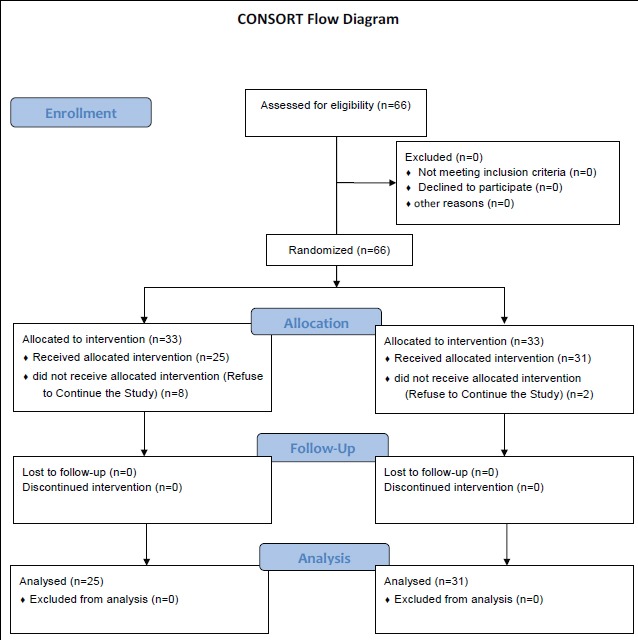
CONSORT Flow Diagram

In this study, eight couples dropped out of the intervention group because of having small children, their husbands’ occupation, not being able to attend the training classes, etc. Also, 2 couples dropped out of the control group for the same reasons listed above. At the end, 25 couples in the intervention group and 31 couples in the control group were enrolled into the study (figure 1).

All the study couples lived in Shiraz, Iran. Both husbands and wives participated in the 1.5 hour education program. The mean age of the wives was 34.6±5.37 and 33.51±4.58 in the intervention and control groups, respectively. The corresponding figures for husbands were 39.12±5.26 and 37.51±4.62, respectively. Besides, 20% of the wives in the intervention group had under diploma degrees, while 80% had high school diplomas or higher degrees. On the other hand, these measures were respectively obtained as 19.4% and 80.6% in the control group. The corresponding figures for the husbands were 28% and 72% in the intervention group and 19.4% and 80.6% in the control group. In this study, 48% of the children in the intervention group were girl and 52% were boy. On the other hand, 61.3% and 38.7% of the control group children were girl and boy, respectively. Moreover, 84% of the intervention group couples had one child and 16% had 2 or more children. In the control group also, 83.9% had one child and 16.1% had 2 or more children. 

Before the intervention, no significant difference was found between the two groups regarding the mean scores of marital conflict and child behavior problem. The two groups were also homogenous concerning the demographic features. 

**Figure 2:**
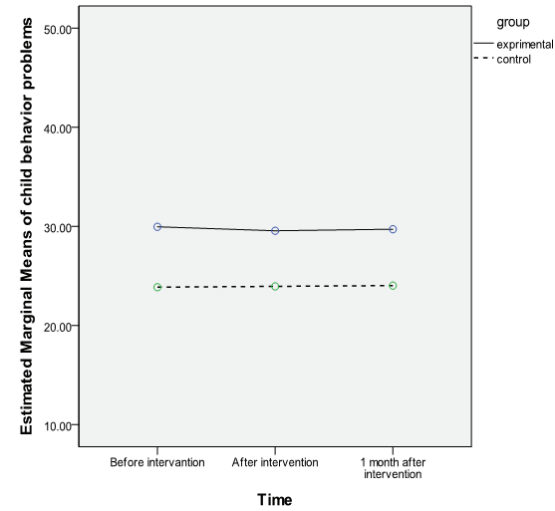
Comparison of changes in child behavior problems based on viewpoints of the wives in the intervention and control groups before, immediately after, and one month after the intervention (the results of repeated measures ANOVA)

Considering the mutual effect of time and group, the results of repeated measures ANOVA revealed a significant improvement in the intervention group wives’ marital conflict in various periods of the study (P<0.001) (table 2). These changes followed a decreasing trend immediately and one month after the intervention. However, no specific difference was seen in the mean scores of child behavior problem before to immediately and one month after the intervention. Thus, it seems that time was not a significant factor in change in this aspect (P=0.6) (table 2 and figure 2).

**Table 2:** Comparison of changes in marital conflict scores in the intervention and control groups’ wives before, immediately after, and one month after the intervention based on repeated measures ANOVA

**Scores**	**Time**	**Before intervention**	**Immediately after intervention**	**One month after intervention**	**P value**		
	**Groups**	**mean±SD**	**mean±SD**	**mean±SD**	**Time **	**Group**	**Time/group**
Marital conflict	Experimental	79±33.14.16	72±92.11.68	71.92±11.45	0.001 *	0.02*	0.001*
	Control	84.29±18.16	84.43±17.26	84.58±16.84			
Child behavior problems	Experimental	29.96±20.82	29.56±20.18	29.72±20.16	0.375	0.210	0.331
	Control	23.87±13.99	23.95±13.66	24.03±13.39			

*Significant at 5% leve

Similarly, the results of repeated measures ANOVA indicated a significant difference in the mean scores of marital conflict from pre-intervention to immediately and one month after the intervention for husbands in the intervention group (P<0.001) (table 2). These changes followed a decreasing trend immediately and one month after the intervention. Nevertheless, no significant difference was seen in the mean scores of child behavior problem before to one month after the intervention (table 2). Regardless of time, no statistically significant difference was found between the intervention and control groups regarding the mean scores of marital conflict (table 3).

**Table 3:** Comparison of changes in the marital conflict scores in the intervention group husbands before, immediately after, and one month after the intervention based on repeated measures ANOVA

**Scores **	**Time**	**Before intervention**	**Immediately after intervention**	**One month after intervention**	**P value**			
	**Groups**	**mean±SD**	**mean±SD**	**mean±SD**	**Time**	**Group**	**Time/group**
Marital conflict	Experimental	78.84±13.40	73.52±12.90	72.40±12.44	0.001 *	0.066	0.001 *
	Control	82.71±17.27	82.45±16.47	82.19±15.89			
Child behavior problems	Experimental	31.72±20.51	31.12±19.63	31.36±19.82	0.355	0.036*	0.184
	Control	22.77±8.65	22.84±8.68	22.90±8.80			

On the other hand, no significant difference was observed in the control group’s mean scores of marital conflict and child behavior problems from before to immediately and one month after the intervention (tables 4 and 5).

**Table 4:** Comparison of the mean scores of marital conflict among the wives in the intervention and control groups based on the results of paired* t* test

**Variable**	**Experimental group**			**Control group**		
	**Before intervention**	**One month after** **intervention**		**Before intervention**	**One month after** **intervention**	
	**mean±SD**	**mean±SD**	**P value**	**mean±SD**	**mean±SD**	**P value**
Marital conflict	79.16±14.32	71.92±11.45	0. 001*	84.29±18.15	84.58±16.83	0.720
Child behavior problems	29.96±20.82	29.72±20.16	0.335	23.87±13.99	24.03±13.39	0.690

*Significant at 5% level

**Table 5:** Comparison of the mean scores of marital conflict among the husbands in the intervention and control groups based on the results of paired t test

**Variable**	**Experimental group**			**Control group**		
	**Before intervention**	**One month after** **intervention**		**Before intervention**	**One month after** **intervention**	
	**mean±SD**	**mean±SD**	**P value**	**mean±SD**	**mean±SD**	**P value**
Marital conflict	78.84±13.40	72.40±12.43	0. 001*	82.70±17.27	82.19±15.89	0.404
Child behavior problems	31.72±20.51	31.36±19.82	0.175	22.77±8.64	22.90±8.80	0.647

*Significant at 5% level

## Discussion

As expected, participating in the training sessions was effective in decreasing the marital conflicts. However, this training was not able to resolve the children’s behavior problems. It is noteworthy that this program was effective for both husbands and wives and could increase their knowledge of beneficial communication and effective relationship with respect to solving their problems in a suitable way.

In fact, this program encouraged the couples to recognize and deal with their conflicts in a more constructive way. The husbands and wives reported more cooperation for solving their problems. Also, they were able to better understand each other’s points of view compared to prior to the training. 

These results are consistent with those of other studies showing that improvement in the couples’ knowledge and skills regarding marital conflicts play an essential role in prevention programs.^11^^,^^21^^,^^22^ However, limited evidence has shown the effectiveness of marital relationship training in the children.

Cummings, Faircloth, and Mitchel evaluated the effects of a brief four-session prevention program on improvement of marital conflicts. The participants were followed up 6 months and one year after the training. The findings indicated that participating in these programs decreased the marital conflicts. Decrease in marital conflicts, in turn, improved the parents’ behaviors and the children’s behavior problems.^23^ Their results were consistent with those of our study, except for improvement in the children’s behavioral problems. Of course, the present study showed a slight reduction in the score of child behavior problems after the intervention, but perhaps a one-month follow-up period was too short for assessing the changes in the children’s behavioral problems. Moreover, it is rational to predict that changes in behavioral problems occur with delay after learning the effective patterns of communication.

One study examined the effects of communication skill training on improvement of parenting roles and social skills in children. In that study, marital relationship training was performed during six sessions and the results indicated the effectiveness of this type of training.^24^

Another study assessed the impact of the communication program developed by Karahan on passive conflict tendencies among the married couples. In that study, 28 married couples, 14 in the control and 14 in the intervention group, were assessed at pre-test, post-test, and follow-up periods. The results showed that the tendency of passive conflict was lower among the married couples who had attended the program in comparison to those who had not. In addition, no significant difference was found between the pre-test and post-test scores in the control group. Follow-up examination was also conducted three and six months after the program had finished and the results showed that the changes were permanent.^25^

In many studies, it has been reported that unresolved conflicts can stop the empathy and decrease the cooperation between the couples.^26^^,^^27^ As a result, it can be claimed that participation in these programs can cause the couples to achieve new and functional communicational skills and also to be able to solve their problems in a more cooperative way.

Moreover, Sardogan and Karahan studied the effects of training a group of couples regarding human relation skills through ten interventional sessions. The results showed an improvement in the couples’ marital adjustment.^28^

Furthermore, Epstein and Baucom evaluated the effects of the cognitive behavioral approach on marital conflicts by considering the correlation between the behavioral approach and cognitive strategies. These common strategies are avoiding conflicts, ending them, and examining their consequences. Hence, when the couples’ knowledge of conflicts increases and they learn how to resolve the conflicts and problems, their relationship will improve.^29^ As shown in the current study, the couples’ marital conflicts improved after learning and performing these skills in real life situations.

Our results are in agreement with those of most other studies confirming the effectiveness of training courses and their long lasting benefits. These training courses should be held before and in the initial stages of marriage to prevent the emerging conflicts. Also, preventive programs on marital distress and conflict resolution may be more effective and economical compared to the programs for remediation of marital distress.^29^

Overall, these findings support the fact that increasing the couples’ knowledge of the communications skills can result in improvement of their marital relationships. Nonetheless, the other hypothesis regarding the reduction of children’s behavioral problems did not come true. Yet, both wives and husbands expressed that their communication with their children improved by improvement in their relationships with each other. They also reported more satisfaction with their relationships. Thus, we can conclude that when the couples feel more satisfied with their marriage, they will interact better with their children. However, as mentioned before, it may take long to be able to see these effects on the children’s behavioral problems.

Of course, the scores obtained from the CBCL might have resulted from other reasons than the couples’ relationships, including problems with schools or peer pressure. There is also a possibility that the parents do not correctly communicate with their children and the problems result from inappropriate performance of parenting roles.

One of the limitations of our study was that our follow-up period was too short to be effective in the children’s behavioral problems. In fact, this trend is continuous and significant changes are expected to be noted over time. Therefore, future studies with larger sample sizes and longer follow-up periods are needed to confirm these results. It would also be beneficial to study the relationship between the participants’ personal characteristics and the effectiveness of interventional programs. For example, although the knowledge of the participants might increase during the intervention, they might still have low self-esteem regarding their ability to cope with their different viewpoints on various matters and handling the familial conflicts.

## Conclusion

Communication skill training was effective in reducing marital conflicts and improving communication patterns. Also, it showed a slight reduction in the score of child behavior problems after the intervention. However, this reduction wasn’t statisticaly significant. Parents stated that after these training sessions, their behavior with their children has been improved as well. Therefore, this method can be recommended for the couples who have marital and parent-child problems. 
